# Supplementary material for: Arachidonic acid promotes the binding of 5-lipoxygenase on nanodiscs containing 5-lipoxygenase activating protein in the absence of calcium-ions
Source: PLoS One. 2020 Jul 9;15(7):e0228607. doi: 10.1371/journal.pone.0228607 (PMC7347166; doi:10.1371/journal.pone.0228607)
Supplement: S1 File — Results from representative examples of native gel electrophoresis in Figs 1 and 2D classifications of negatively stained (uranyl formate) samples in Fig 2. (PDF) [file pone.0228607.s001.pdf]

**S2 File**: Figures 1 and 2 containing the results from representative examples of native gel electrophoresis and negative staining (uranyl formate) of the complexes.

**Figure 1. The presence of AA is sufficient to induce binding of 5LO on the FLAP-nanodiscs.**

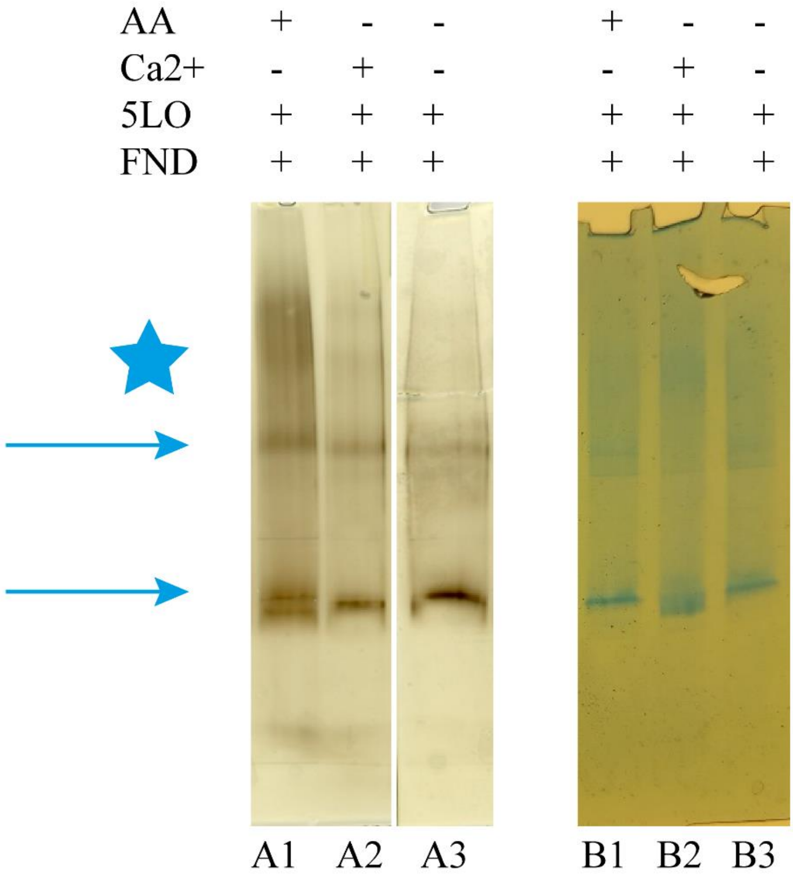


**Figure 1. Formation of the complex 5LOFND, in the presence of either AA or $Ca^{2+}$-ions.** The gels shown contained neither $Ca^{2+}$ nor AA in the buffers during the gel-runs. Both gels were 4-16% Tris glycine Native page (A) or Blue Native page (B). The columns of + or – indicates the substances present in the sample mixture loaded in corresponding gel lane. The star indicates the level where bands show which may correspond to 5LOFND formed by the presence of either AA or $Ca^{2+}$. To make the figure, relevant lanes were cut in the original gel images and rearranged in (A). A section was cut out from the original gel image in (B), see S1_Raw_files.pdf.

For the two gels in Figure 1, the sample preparations were different to test if the order of addition could have effects on 5LOFND complex-formation. For the gel in Fig. A, a specific amount of substance was added to the FND solution and incubated for 5 minutes before addition of 5LO in equimolar amounts to FND (0.6 µM) and left to react for another 5 minutes. The amount of substance added provided concentrations of 50 µM AA (lane A1) or 1 mM $Ca^{2+}$ (lane A2) both in the presence of 0.5 mM EDTA. This sample preparation is similar to that used for activity measurements earlier (Kumar *et al.*, 2017). Under these conditions of substance additions the 5LOFNDAA complex formed (Lane A1) indicated by bands at higher levels (star) than FND, the FND band (upper arrow) was slowed down and a split 5LO band appears (lower arrow). The bands representing the $Ca^{2+}$ induced complex appear weak (Lane A2, star-level) under these conditions.

A 5LOFNDCa complex was formed however, using another order of addition. For the gel in Fig. B, the samples were made by placing drops containing either AA, $Ca^{2+}$ or just buffer on the wall of an Eppendorf tube followed by rapid centrifugation to mix the substance additions into the pre-mixed equimolar 5LO and FLAP solutions. The final concentrations are the same as above, 0.6 µM protein, 50 µM AA or 1 mM $CaCl_2$. In Lane B2, the rapid addition of $Ca^{2+}$ appears to have induced the formation of a 5LOFNDCa complex at a higher Mw (star) at the expense of the FND for which the band has almost disappeared (upper arrow). In addition, the 5LO band appears split (lower arrow, Lane B2). Lane B1, where the sample should contain the AA complex, displays a very weak band at higher Mw (Lane B1), the 5LO band is not split and the bands representing FND and 5LO have intensities similar to the control in lane B3.

**Figure 2. 2D analysis of UF stained samples.**

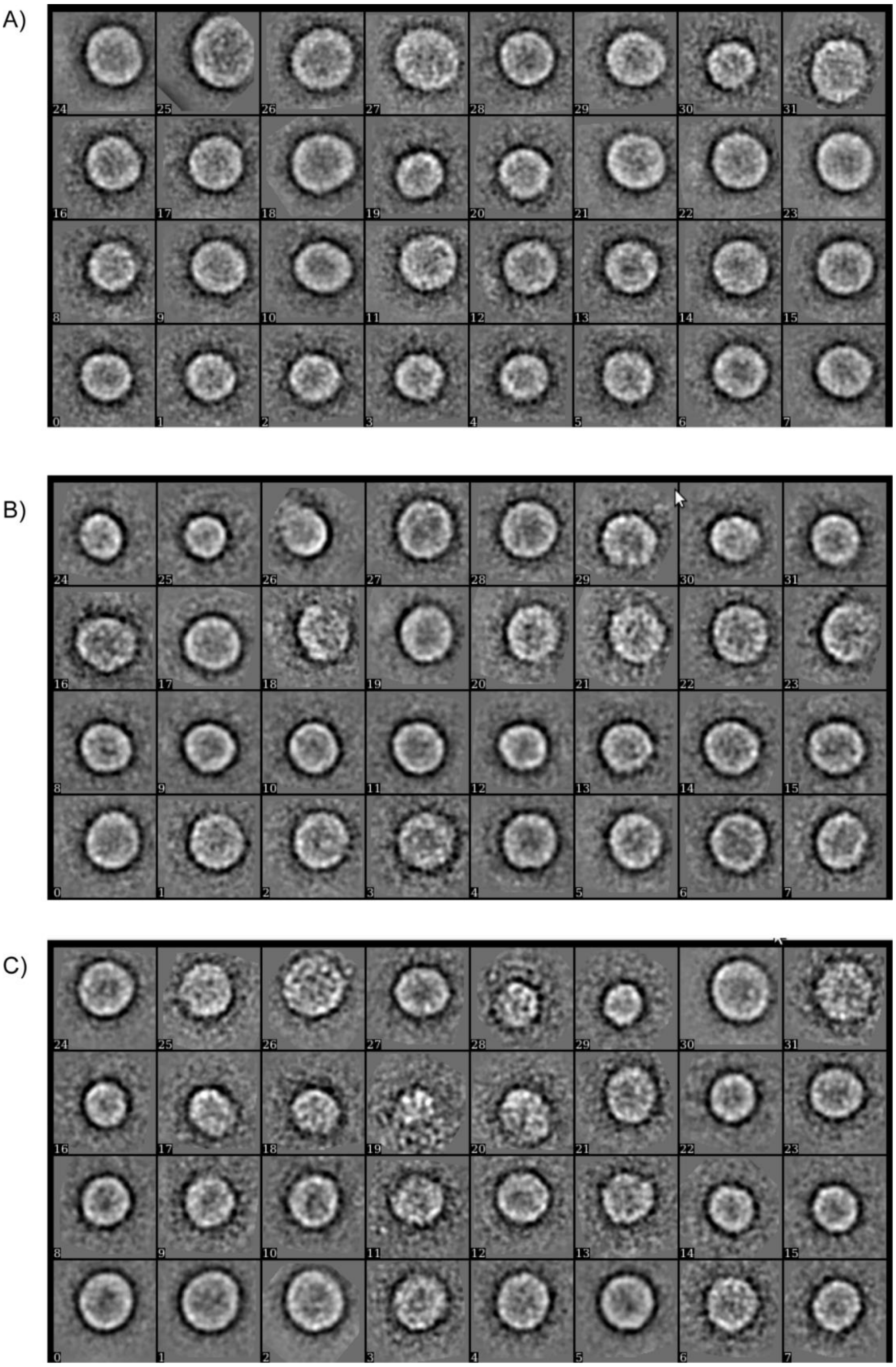

**Figure 2. 2D analysis of UF stained samples.** 2D-class averages of data processed by EMAN2 (Tang *et.al*. 2007) for UF-stained samples: A) FND B) 5LOFNDAA and C) 5LOFNDCa. Particle sets were 3081 (FND), 3705 (5LOFNDAA) and 3201 (5LOFNDCa). The box side length is 30.1 nm.

**References:**


Kumar R.B., Zhu L., Hebert H. & Jegerschöld C. (2017): Method to visualize and analyse membrane interacting proteins by transmission electron microscopy. *J. Vis. Exp.* (), e55148, doi: 10.3791/55418.

Tang G., Peng L., Baldwin P.R., Mann D.S., Jiang W., Rees I. & Ludtke S.J. (2007): EMAN2: an extensible image processing suite for electron microscopy. *J Struct Biol.* 157, 38-46
