# Supplementary material for: Arachidonic acid promotes the binding of 5-lipoxygenase on nanodiscs containing 5-lipoxygenase activating protein in the absence of calcium-ions
Source: PLoS One. 2020 Jul 9;15(7):e0228607. doi: 10.1371/journal.pone.0228607 (PMC7347166; doi:10.1371/journal.pone.0228607)
Supplement: S1 Raw Images — (PDF) [file pone.0228607.s002.pdf]

S1_Raw_images

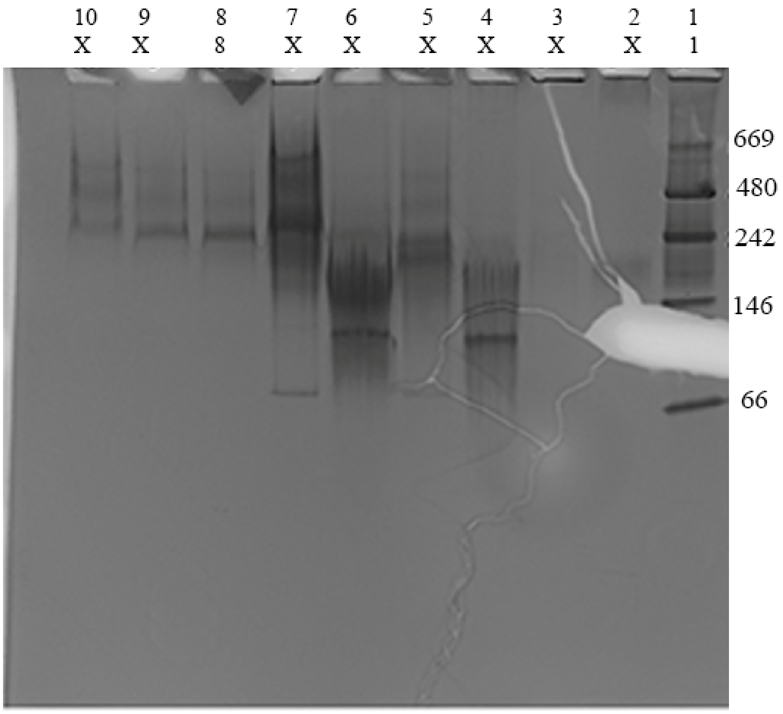

Main text Fig 1A :
The marker in Lane 1 is to the right in both gels.

Purified nanodiscs containing FLAP shows in Lane 8 in the original gel and to the left of the marker in Main text Fig1A.

The original gel shows most steps in the reconstitution procedure of FLAP into nanodiscs.

Lanes 8-10 are elution fractions from a size-exclusion column purification.

The gel is a 4-20 % tris-glycine native PAGE.

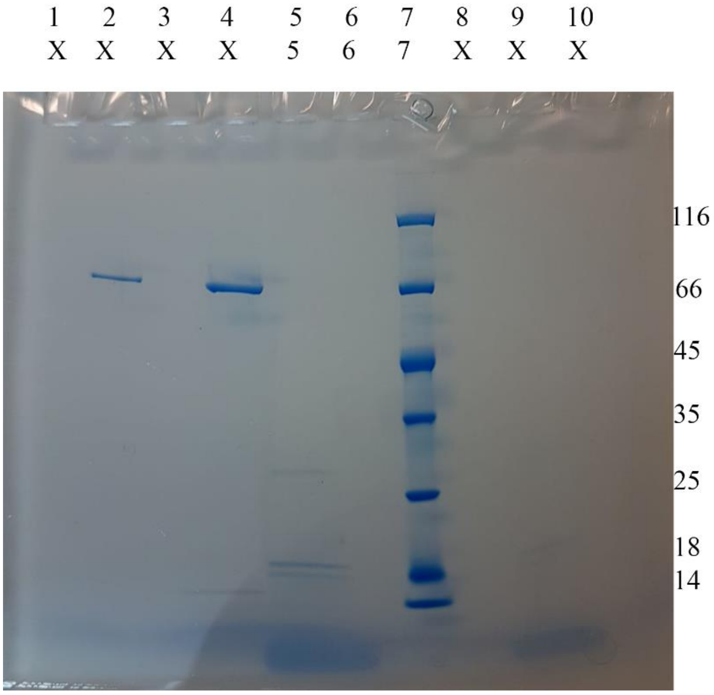

Main text Fig 1B :
The marker in Lane 7 here is to the right in Main Fig1B.

Lane 6 is empty.
Lane 5 comprises nanodiscs containing FLAP. Denaturing by SDS results in bands from MSP1E3D1, ca 25 kDa, and FLAP-C-His6, 18.5 kDa. The band at ca 18 kDa could indicate a breakdown product of FLAP.

Cropped parts of Lanes 5-7 are present in Main text Fig1B.

Lanes 2 and 4 shows 5LO at different concentrations.
Lanes 1,3,6,8 and 10 are empty.

The gel is a 10-20 % tris-glycine SDS PAGE

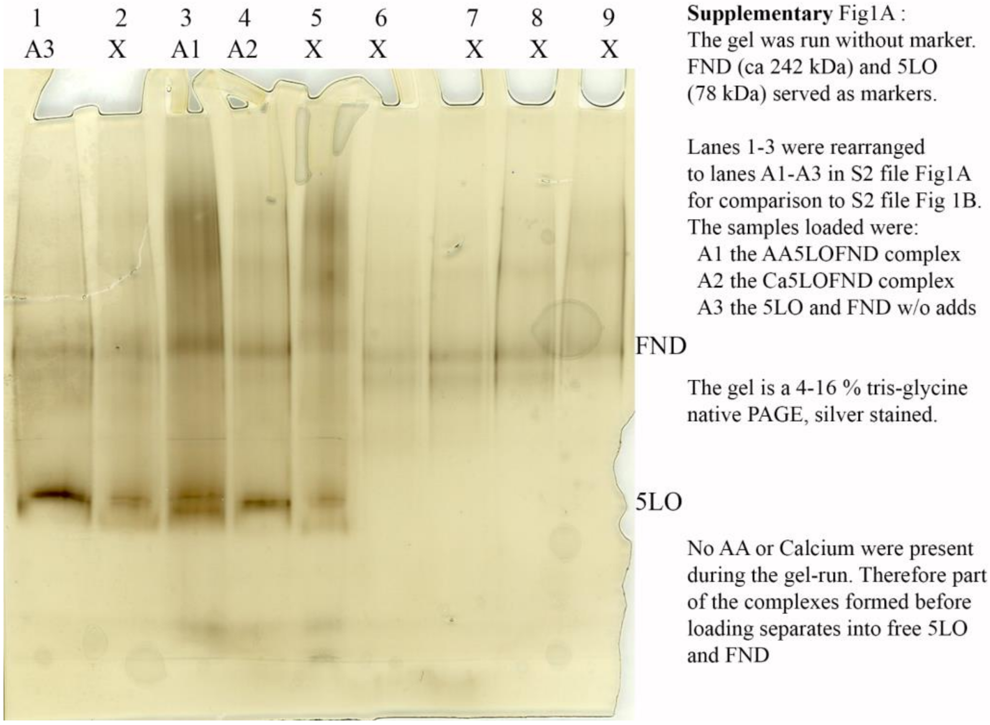

Supplementary Fig1A :
The gel was run without marker.
FND (ca 242 kDa) and 5LO
(78 kDa) served as markers.

Lanes 1-3 were rearranged
to lanes A1-A3 in S2 file Fig1A
for comparison to S2 file Fig 1B.
The samples loaded were:
A1 the AA5LOFND complex
A2 the Ca5LOFND complex
A3 the 5LO and FND w/o adds

The gel is a 4-16 % tris-glycine
native PAGE, silver stained.

No AA or Calcium were present
during the gel-run. Therefore part
of the complexes formed before
loading separates into free 5LO
and FND

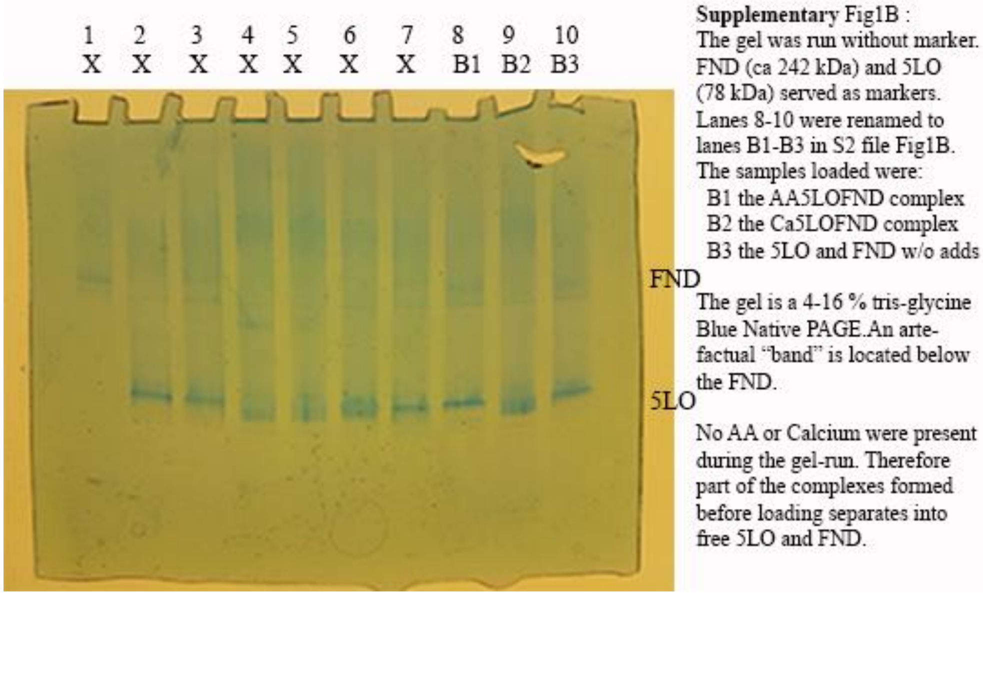

Supplementary Fig1B :
The gel was run without marker.
FND (ca 242 kDa) and 5LO
(78 kDa) served as markers.
Lanes 8-10 were renamed to
lanes B1-B3 in S2 file Fig1B.
The samples loaded were:
B1 the AA5LOFND complex
B2 the Ca5LOFND complex
B3 the 5LO and FND w/o adds

The gel is a 4-16 % tris-glycine
Blue Native PAGE.An arte-
factual "band" is located below
the FND.

No AA or Calcium were present
during the gel-run. Therefore
part of the complexes formed
before loading separates into
free 5LO and FND.

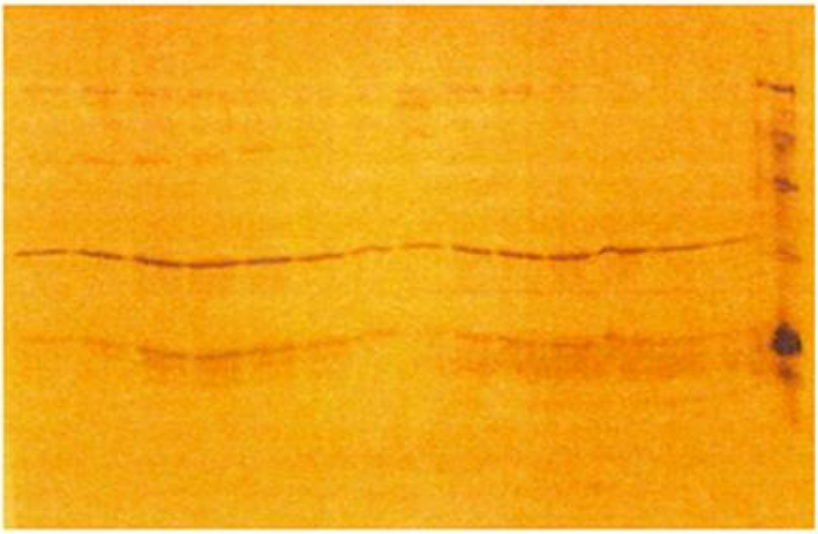

Main text Fig 3:

The original gel is presented the main text in Fig3.
